# Compositional and Proteomic Analyses of Genetically Modified Broccoli (*Brassica oleracea* var. *italica*) Harboring an Agrobacterial Gene

**DOI:** 10.3390/ijms150915188

**Published:** 2014-08-28

**Authors:** Mao-Sen Liu, Miau-Hwa Ko, Hui-Chun Li, Shwu-Jene Tsai, Ying-Mi Lai, You-Ming Chang, Min-Tze Wu, Long-Fang O. Chen

**Affiliations:** 1 Institute of Plant and Microbial Biology, Academia Sinica, Nankang, Taipei 11529, Taiwan; E-Mails: d89226001@ntu.edu.tw (M.-S.L.); ayamama@gate.sinica.edu.tw (H.-C.L.); ymlai@gate.sinica.edu.tw (Y.-M.L.); 2 Department of Anatomy, School of Medicine, China Medical University, Taichung 40402, Taiwan; E-Mail: mhko@mail.cmu.edu.tw; 3 2nd Unit, Taiwan Agricultural Research Institute, Wufeng, Taichung 41362, Taiwan; E-Mails: SJTsai@tari.gov.tw (S.-J.T.); wu@tari.gov.tw (M.-T.W.); 4 Department of Bioindustry Technology, Dayeh University No. 168, University Rd., Dacun, Changhua 51591, Taiwan; E-Mail: cym2390@yahoo.com.tw

**Keywords:** allergen, *Brassica oleracea* var. *italica*, broccoli, proteomics, glucosinolate, *isopentenyltransferase* (*ipt*)

## Abstract

Previously, we showed improved shelf life for agrobacterial *isopentenyltransferase* (*ipt*) transgenic broccoli (*Brassica oleracea* var. *italica*), with yield comparable to commercial varieties, because of the protection mechanism offered by molecular chaperones and stress-related proteins. Here, we used proximate analysis to examine macronutrients, chemical and mineral constituents as well as anti-nutrient and protein changes of *ipt*-transgenic broccoli and corresponding controls. We also preliminarily assessed safety in mice. Most aspects were comparable between *ipt*-transgenic broccoli and controls, except for a significant increase in carbohydrate level and a decrease in magnesium content in *ipt*-transgenic lines 101, 102 and 103, as compared with non-transgenic controls. In addition, the anti-nutrient glucosinolate content was increased and crude fat content decreased in inbred control 104 and transgenic lines as compared with the parental control, “Green King”. Gel-based proteomics detected more than 50 protein spots specifically found in *ipt*-transgenic broccoli at harvest and after cooking; one-third of these proteins showed homology to potential allergens that also play an important role in plant defense against stresses and senescence. Mice fed levels of *ipt*-transgenic broccoli mimicking the 120 g/day of broccoli eaten by a 60-kg human adult showed normal growth and immune function. In conclusion, the compositional and proteomic changes attributed to the transgenic *ipt* gene did not affect the growth and immune response of mice under the feeding regimes examined.

## 1. Introduction

Transgenic technologies lead to genetically modified (GM) crops and promote food quantity and quality [1,2]. GM crops with pest and disease resistance, stress tolerance, edible vaccines, and added vitamins and nutrients are beneficial for humans [3,4]. For example, GM corn containing a gene that encodes for *Bacillus* *thuringiensis* (Bt) toxin acquired resistance to certain insect herbivores and produced a lower level of mycotoxin [5], which in turn reduced the use of pesticides and improved human and animal health. The global area of production of GM crops has reached 160 million hectares in the last 15 years [6]. Despite the many advantages of GM crops, their acceptance in many countries is controversial, especially in Europe [7]. Besides environmental and ecological issues [8], whether GM crops are safe for human health is a major concern [7].

Most crops naturally produce allergens, toxins and anti-nutritional substances [9]. Conventional breeding by crossing crops and transgenic breeding by introducing a foreign gene into a plant can both modify the genome and may unintentionally change the composition of these substances in a crop [9]. High-level uptake of these substances might have adverse effects on animal and human growth and health; for example, antigens may cause allergies, toxins may cause intoxications, and anti-nutritional substances prevent nutrient absorption.

Proteins and peptides in crops may possess allergen, toxin and anti-nutritional activities. Allergens in food are almost always proteins [10]. Also, food lectins and protease inhibitors are potential anti-nutritional or toxic components that have adverse effects on human health [11,12,13]. Besides proteins, glucosinolates, secondary plant metabolites, are common anti-nutritional substances in *Brassica* crops, including broccoli [14]. Although glucosinolates and their breakdown products may have anti-cancer activities [15], high-level uptake can delay the growth of animals [16].

Breeders have used many strategies including non-targeted proteomics and targeted anti-nutrient analysis to identify the unintended effects of GM food [17,18,19]. Besides the analysis of substantial equivalence by examining the similarities and differences between GM crops and their non-GM counterparts, further analysis of GM crops in terms of animal feeding can help in evaluating the safety of GM crops for human health [17,19].

Previously, we demonstrated an improvement in delaying postharvest senescence without affecting yield with transgenic *isopentenyltransferase* (*ipt*) broccoli derived from fifth-generation inbred (selfed-T_5_) progeny [20,21]. The transgenic *ipt* mimics the action of N^6^-benzylaminopurine BA that delays postharvest senescence by accumulation of molecular chaperones and stress-related proteins [22,23]. However, information on the substantial equivalence of this GM crop is absent. In this report, we used proximate analyses of macronutrients, chemical and mineral constituents as well as the level of glucosinolates, and changes in the level of endogenous proteins of *ipt*-transgenic broccoli and corresponding controls. We found altered levels of magnesium and carbohydrates and some differentially expressed endogenous proteins with homology to protein allergens in *ipt*-transgenic broccoli. We also studied the physiologic effects and immune responses in mice fed 2 levels of *ipt*-transgenic broccoli. The results indicated that the compositional and proteomic changes do not reach a threshold to affect growth or induce an immune response in mice under 2 regimes of feeding.

## 2. Results and Discussion

### 2.1. Proximate Analysis of Macronutrients, Chemical and Mineral Constituents

We performed proximate analysis of the parental line green king (GK), the GK inbred line 104 and the transgenic inbred lines 101, 102 and 103 (Table 1). Despite some variations between different lines, overall, we found no significant or consistent variation in water, crude protein, crude fiber or ash content between transgenic lines and their counterparts, line 104 and the parental line GK. However, crude fat content was lower, by about 10%, in inbred transgenic lines, 101, 102 and 103, and control line 104 than the parental line GK. In addition, carbohydrate content was increased about 9% to 22% in *ipt*-transgenic lines as compared with the control lines.

**Table 1:** Proximate analysis of broccoli florets.

Line	Water Content (%)	Crude Protein (%)	Crude Fat (%)	Crude Fiber (%)	Ash (%)	CHO (%)
101	85.58 ± 0.21 ^c,d^	36.15 ± 0.86 ^c^	7.16 ± 0.24 ^b^	9.17 ± 0.35 ^b^	8.02 ± 0.25 ^a,b^	39.50 ± 0.53 ^a^
102	85.17 ± 0.39 ^d^	37.73 ± 0.25 ^c^	7.14 ± 0.46 ^b^	8.23 ± 0.35 ^c^	7.86 ± 0.37 ^b^	39.04 ± 0.24 ^a^
103	86.78 ± 0.32 ^a^	40.37 ± 0.43 ^b^	7.22 ± 0.32 ^b^	9.78 ± 0.39 ^a^	7.42 ± 0.16 ^c^	35.20 ± 0.33 ^b^
104	85.81 ± 0.23 ^b,c^	43.49 ± 1.90 ^a^	7.48 ± 0.31 ^b^	8.69 ± 0.21 ^b,c^	7.79 ± 0.17 ^b,c^	32.55 ± 1.78 ^c^
GK	86.20 ± 0.34 ^b^	40.89 ± 1.46 ^b^	8.33 ± 0.37 ^a^	10.16 ± 0.13 ^a^	8.42 ± 0.19 ^a^	32.20 ± 1.14 ^c^

Florets from 5 individuals were mixed as described in the Experimental Section; The data are mean ± SD of 3 experimental repeats; GK, green king; CHO, carbohydrates; Water content is presented on a fresh weight basis and crude protein, fat, fiber ash and CHO are presented on a dry weight basis; Symbols a, b, c and d indicate the differences between samples. Means with the same symbol in a column are not significantly different at *p* < 0.05 whereas those with different symbols are significantly different.

Similar results were shown with chemical constituent analysis (Table 2). The content of ascorbate, titratable acidity, formol nitrogen (formol-N), free sugar and insoluble solid did not significantly differ between transgenic broccoli and the non-transgenic controls, although we observed some variations in ascorbate, free sugar and insoluble solid content between different lines.

Only magnesium content was lower by about 19% to 30% in transgenic lines than in the control lines 104 and GK (Table 3). The content of phosphorus, potassium, calcium, iron, manganese, copper and zinc did not significantly differ between *ipt*-transgenic broccoli and controls. The content of potassium was lower and that of copper, and zinc was higher in line 104 than in transgenic lines and GK. The content of manganese was lower in transgenic lines 102 and 103 (but not line 101) than in the control lines 104 and GK.

**Table 2:** Chemical constituents of broccoli florets.

Line	Ascorbic Acid (%)	Titratable Acidity (%)	Formol-N (%)	Free Sugar (%)	Insoluble Solid (%)
101	0.34 ± 0.06 ^c^	2.00 ± 0.10 ^a^	0.53 ± 0.06 ^a^	9.93 ± 0.97 ^b^	38.11 ± 3.10 ^b^
102	0.56 ± 0.01 ^a^	2.16 ± 0.51 ^a^	0.52 ± 0.04 ^a^	13.14 ± 1.23 ^a^	42.42 ± 2.65 ^a,b^
103	0.46 ± 0.04 ^b^	2.21 ± 0.07 ^a^	0.52 ± 0.01 ^a^	10.37 ± 2.61 ^b^	46.33 ± 1.73 ^a^
104	0.28 ± 0.07 ^c^	2.41 ± 0.11 ^a^	0.69 ± 0.09 ^a^	8.19 ± 0.08 ^b^	44.69 ± 1.62 ^a^
GK	0.33 ± 0.04 ^c^	2.15 ± 0.39 ^a^	0.61 ± 0.15 ^a^	8.67 ± 0.67 ^b^	44.94 ± 2.65 ^a^

Florets from 5 individuals were mixed as described in the Experimental Section; The data are mean ± SD of 3 experimental repeats; The data are presented on a dry weight basis; The titratable acidity is expressed in terms of citric acid; The free sugar content is expressed in terms of glucose; Symbols a, b and c indicate the differences between samples. Means with the same symbol in a column are not significantly different at *p* < 0.05 whereas those with different symbols are significantly different.

**Table 3:** Mineral content of broccoli florets.

Line	P	K	Ca	Mg	Fe	Mn	Cu	Zn
101	591 ± 27 ^b^	3391 ± 164 ^a^	437 ± 62 ^b^	195 ± 17 ^b^	9.24 ± 1.06 ^a^	4.16 ± 0.16 ^a,b^	0.51 ± 0.05 ^b^	5.53 ± 0.32 ^b^
102	532 ± 45 ^a,b^	3177 ± 78 ^a,b^	518 ± 18 ^a,b^	195 ± 6 ^b^	10.60 ± 7.05 ^a^	2.83 ± 0.03 ^b^	0.53 ± 0.01 ^b^	5.58± 0.36 ^b^
103	496 ± 18 ^b^	3037 ± 47 ^b^	512 ± 42 ^a,b^	192 ± 10 ^b^	7.84 ± 0.62 ^a^	3.33 ± 0.16 ^b^	0.44 ± 0.02 ^c^	5.44 ± 0.16 ^b^
104	532 ± 40 ^a,b^	2643 ± 326 ^c^	576 ± 96 ^a^	238 ± 28 ^a^	9.53 ± 0.70 ^a^	4.36 ± 0.39 ^a^	0.62 ± 0.05 ^a^	6.97 ± 0.49 ^a^
GK	605 ± 1 ^a^	3421 ± 198 ^a^	613 ± 27 ^a^	264 ± 2 ^a^	7.75 ± 0.59 ^a^	4.60 ± 0.95 ^a^	0.51 ± 0.03 ^b,c^	5.36 ± 0.27 ^b^

Florets from 5 individuals were mixed as described in Experimental Section; The data are mean ± SD of 3 experimental repeats; The data are presented on a dry weight basis of mg/100 g; Symbols a, b, and c indicate the differences between samples. Means with the same symbol in a column are not significantly different at *p* < 0.05 whereas those with different symbols are significantly different.

The higher carbohydrate content in *ipt*-transgenic than control lines might be attributed to downregulation of genes and proteins involved in carbohydrate metabolism [23], in turn modulating their contents in *ipt*-transgenic broccoli. Except for the carbohydrate and magnesium content, proximate analysis of the other constituents did not reveal significant differences between *ipt*-transgenic broccoli and their control lines.

### 2.2. Proteins Specifically Detected in Ipt-Transgenic Broccoli at Harvest and after Cooking

**Figure 1:**
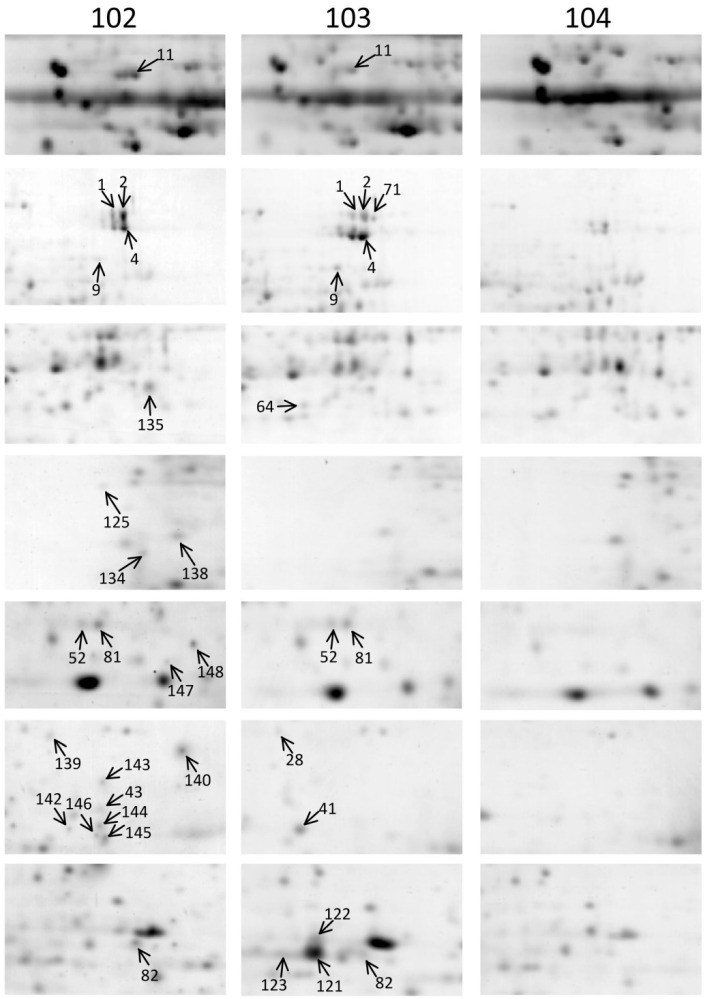
Protein spots specifically detected in *isopentenyltransferase* *(ipt)*-transgenic broccoli at harvest. Number indicates protein spots specifically detected in the 2 *ipt*-transgenic lines 102 or 103 at harvest as compared with the control 104.

**Figure 2:**
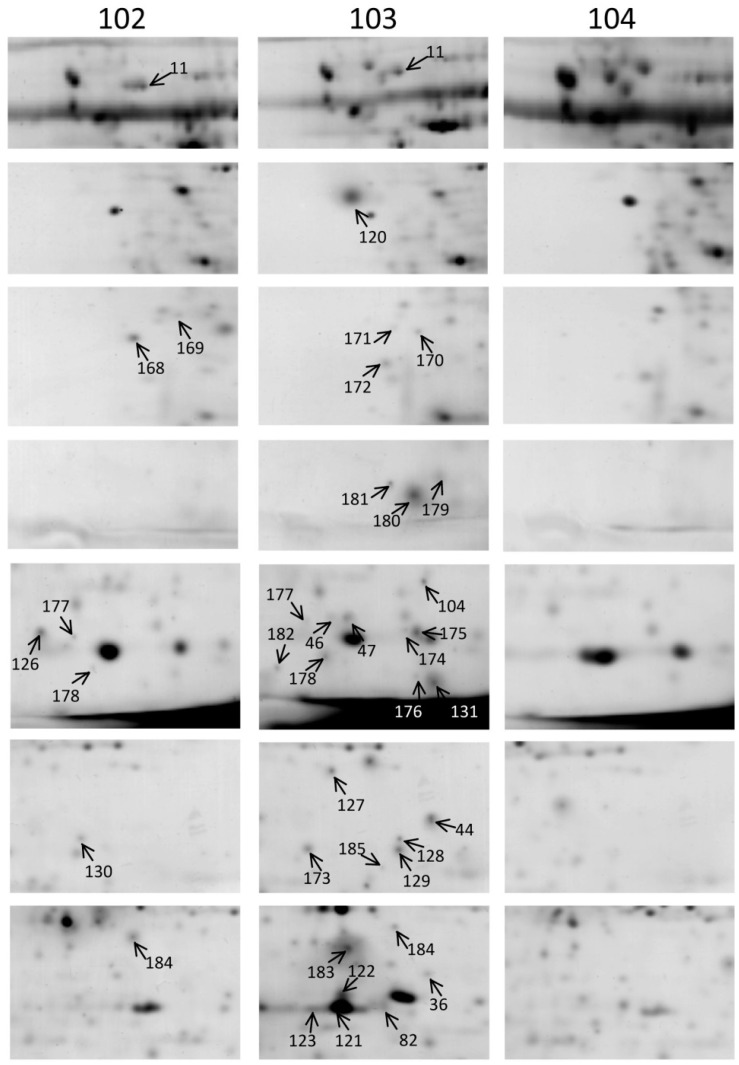
Protein spots specifically detected in *isopentenyltransferase* *(ipt)*-transgenic broccoli after cooking. Number indicates protein spots specifically detected in the 2 *ipt*-transgenic lines 102 or 103 after cooking as compared with the control 104.

To determine whether *ipt*-transgenic broccoli produced differentially expressed endogenous or novel proteins caused by T-DNA insertion that may have negative effects on human health, 2D gel electrophoresis (2-DE) was used to compare protein profiles between non-transgenic control and 2 independent *ipt*-transgenic broccoli lines, 102 and 103, at harvest and after cooking. The florets from 3 individuals of each line of each treatment were mixed in equal weight for protein extraction and analysis. The 2-DE of each sample was performed twice. The protein profile was analyzed in 12 2D gels. Both times produced protein spots specifically detected for *ipt*-transgenic lines as compared with control lines (Figure 1, Figure 2 and S1, Table 4 and S1). Because we aimed to find *ipt*-transgenic-related harmful proteins, we focused on proteins specifically detected in the *ipt*-transgenic broccoli, line 102 or 103, as compared with the control. In general, we detected >500 protein spots on each gel, and 59 were detected only on *ipt*-transgenic broccoli gels. Of the 59 protein spots specifically detected in *ipt*-transgenic broccoli, 24 were detected at harvest, 30 after cooking and 5 at both times. A total of 8 spots (1, 2, 4, 9, 11, 52, 81 and 82) and 4 spots (11, 177, 178 and 184) were detected in *ipt*-transgenic lines at harvest and after cooking, respectively. More protein spots were specifically detected for line 102 than 103 at harvest (14 *vs.* 7), and for line 103 than 102 after cooking (28 *vs.* 4) (Figure 1, Figure 2 and S1).

Modification in the genome of transgenic plants might cause broad changes in transcriptome, proteome and metabolome. Lines 102 and 103 are 2 independent *ipt*-transgenic lines with different insertion sites [21]. Their different genomic modifications resulted in changed protein composition and physiologic features in that postharvest senescence is delayed more with line 103 than line 102 [21].

Heat denatures proteins and changed the protein composition in broccoli after cooking for 5 min. The difference in protein composition in the 2 *ipt*-independent lines implies their different responses to cooking and explains the changes in number of proteins specifically detected after cooking.

### 2.3. Identification of Proteins Specifically Detected in Ipt-Transgenic Broccoli by LC–ESI-MS/MS

LC-ESI-MS/MS detected 49 of the 59 protein spots specifically detected in *ipt*-transgenic broccoli at harvest or after cooking. With our criteria for identifying protein spots, 10 spots (36, 125, 168, 171, 172, 176, 179, 180, 181 and 182) could not be identified. Therefore, 49 spots were the best hits to proteins showing homology to Arabidopsis proteins (Table 4 and S1).

Many protein spots showed hits to the same protein candidate or homology to the same *Arabidopsis* protein (Table 4 and S1), which implies that they may belong to 1 gene family, post-translationally modified in broccoli or the fragmentation of one protein during experimental processes. Spots 1 and 4 both showed hits to *Arabidopsis* 10-formyltetrahydrofolate synthase. Spots 41 and 43 were the best hits for *Arabidopsis* and *B. napus* proteins, respectively, but were homologous to the same *Arabidopsis* protein. Spots 46, 47 and 147 and spots 131, 174, 175 and 177 all showed hits to ribulose 1,5-bisphosphate carboxylase/oxygenase of *B. napus*, although with different protein accession numbers. Spots 52 and 81 showed hits to an unknown protein of *B. rapa*; spots 82 and 123 to putative type peroxiredoxin of *B. rapa* subsp. *pekinensis*; spots 104, 144, 145, 146 and 185 to *Arabidopsis* cystathionine β-synthase family proteins with different protein accessions numbers; spots 121 and 129 to water-soluble chlorophyll protein of *B. oleracea* var. *botrytis*; spots 122, 173 and 184 to *B. oleracea* trypsin inhibitor; spots 127 and 139 to *B. rapa* hydrogen-transporting ATP synthase; and spots 128 and 130 to an unknown protein of *B. napus*. The 49 protein spots identified by LC–ESI-MS/MS represented 30 proteins.

A further BLAST search of the Allergen Database for Food Safety database with proteins specifically detected in *ipt*-transgenic broccoli (Table 4 and S1) revealed 17 protein spots with homology to allergens in 5 allergen categories: Areo Fungi, Areo Plant, Areo Insect, Food Plant and Contact (Table 5), including Alt a 4, Hev b 10.0101, Mal f 2, Bet v 7, Mala f 4, Mal f 3, Gly m, Car b 1 and Tyr p allergens. The identity between identified proteins and the defined allergens ranged from 25% to 86%. We detected Alt a 4 and Hev b 10.0101 allergens in *ipt*-transgenic broccoli at harvest and after cooking but Mal f 2 allergen only at harvest and Bet v 7 allergen only after cooking. However, we detected Mala f 4 and Mal f 3 specifically in transgenic line 103 and Car b 1 and Tyr p in line 102. Even in non-transgenic broccoli, we detected proteins with homology to protein allergens, for example, the unnamed protein (*B. napus*), malate dehydrogenase and glyoxalase I (*B. juncea*) [23]. Whether these proteins specifically identified in *ipt*-transgenic broccoli possess allergenic activities still needs further evaluation.

Protein allergens in foods may be heat-stable or -labile, and new ones may be generated during food processing [24]. The possible protein allergens, Mn superoxide dismutase (MnSOD; spot 28), antioxidant/peroxidase (spots 41 and 43), mitochondrial malate dehydrogenase 1 (spot 64) and calmodulin (spot 143), were detected only at harvest, which implies that they are likely heat-labile. The detection of protein disulfide isomerase (PDI; spot 11), type 2 peroxiredoxin (spots 82 and 123), water-soluble chlorophyll protein (spot 121) and trypsin inhibitor (spot 122) both at harvest and after cooking in *ipt*-transgenic broccoli indicated that they likely have heat-stable properties. Other possible protein allergens detected only after cooking showed heat tolerance during cooking: unnamed protein (spots 44, 128 and 130), water-soluble chlorophyll protein (spot 129), trypsin inhibitor (spots 173 and 184) and bet v I allergen family protein (spot 126). The heat-stable protein allergens and those not properly digested and denatured during ingestion and digestion in mammalian digestive systems are most likely associated with a hypersensitivity disorder of the immune system [25].

**Table 4:** Proteins specifically identified in *isopentenyltransferase (ipt)*-transgenic broccoli.

Spot	Accession Best-Hit Protein_ *Arabidopsis* Homolog	*M*r/p*I* Theor. ^d^	*M*r/p*I* Exp. ^e^	Protein Score ^f^	Sequence Coverage (%)	Peptides from MS/MS ^g^
1 ^a^	gi|5921663 10-formyltetrahydrofolate synthetase ( *Arabidopsis thaliana*)_At1g50480	68.3/6.3	66.0/7.1	503	21	18
4 ^a^	gi|18403095 10-formyltetrahydrofolate synthetase ( *A. thaliana*)_At1g50480	67.8/6.3	63.0/7.1	40.3	8	5
2 ^a^	gi|2642429 putative poly(A) binding protein ( *A. thaliana*)_At2g23350	72.0/6.4	66.0/7.2	164	14	13
9 ^a^	gi|30694221 mitochondrial/lipoamide dehydrogenase 1 ( *A. thaliana*)_At1g48030	54.0/7.1	53.0/6.8	262.4	55.4	50
11 ^c^	gi|77999357 protein disulfide isomerase ( *Brassica carinata*)_At1g77510	55.7/4.9	54.0/5.3	330.3	64.8	74
28 ^a^	gi|148515008 Mn superoxide dismutase ( *Eutrema halophilum*)_At3g10920	25.5/9.1	23.0/6.2	50.3	26	8
41 ^a^	gi|18397457 antioxidant/peroxidase ( *A. thaliana*)_At3g06050	21.4/9.3	16.0/6.4	80.3	40.8	14
43 ^a^	gi|227247698 unnamed protein product ( *B. napus*)_At3g06050	21.6/9.3	18.0/6.6	80.3	33.8	16
44 ^b^	gi|219900341 unnamed protein product ( *B. napus*)_At4g38740	18.3/8.2	20.0/8.4	60.2	24.4	12
46 ^b^	gi|296784927 ribulose 1,5-bisphosphate carboxylase/oxygenase (RUBISCO) ( *B. rapa* subsp. *Chinensis*)_At5g38430	20.3/8.1	14.0/6.2	80.3	37.6	14
47 ^b^			14.5/6.3	60.2	39.8	8
147 ^a^			14.0/6.9	68.3	44.2	13
131 ^b^	gi|119720808 ribulose bisphosphate carboxylase ( *B. rapa*)_At5g38430	18.4/8.1	9.3/7.1	30.2	24.7	3
174 ^b^			11.0/6.5	68.3	49.4	10
175 ^b^			11.0/6.6	60.3	45.7	9
177 ^b^			11.6/6.1	80.3	44.4	13
52 ^a^	gi|119720802 unknown ( *B. rapa*)_At3g17020	17.8/6.1	11.0/6.9	78.3	33.1	14
81 ^a^			14.1/6.4	78.3	43.6	12
64 ^a^	gi|2497857 mitochondrial malate dehydrogenase 1 ( *B. rapa*)_At1g53240	35.7/9.0	30.0/6.4	186.3	46	73
71 ^a^	gi|7076772 dynamin-like protein 4 (ADL4) ( *A. thaliana*)_At3g60190	69.8/7.2	67.0/7.2	200	18	13
82 ^c^	gi|4928472 type 2 peroxiredoxin ( *B. rapa* subsp. *pekinensis*)_At1g65980	17.4/5.3	15.8/5.9	120.4	53.7	24
123 ^c^			16.7/5.5	118.3	58	20
104 ^b^	gi|15238284 cystathionine β-synthase (CBS) family protein ( *A. thaliana*)_ At5g10860 gi|297811195 CBS family protein (*A. thaliana*)_ At5g10860	22.7/9.4 22.7/9.4	13.7/6.9	78.3	16.5	14
144 ^a^			14.8/6.6	40.3	20.9	9
145 ^a^			14.3/6.6	124.3	20.9	26
146 ^a^			14.3/6.5	98.2	20.9	24
185 ^b^			16.0/7.4	40.2	20.9	5
120 ^b^	gi|15242351 alpha-1,4-glucan-protein synthase (UDP-forming) ( *A. thaliana*)_At5g15650	40.9/5.7	35.0/3.7	212.3	43.9	35
121 ^c^	gi|3551243 water-Soluble Chlorophyll Protein ( *B. oleracea* var. *botrytis*)_At1g72290	23.5/8.4	16.7/5.7	100.3	23.9	114
129 ^b^			17.7/8.0	30.2	16.1	4
122 ^c^	gi|183988826 trypsin inhibitor ( *B. oleracea*_At1g73260	17.5/8.3	17.0/5.7	130.3	71.1	70
173 ^b^			16.0/6.3	30.3	27.7	7
184 ^b^			21.7/5.9	34	40.26	7
126 ^b^	gi|157849664 bet v I allergen family protein ( *B. rapa*)_At1g24020	17.2/5.2	11.6/6.0	60.3	31	10
127 ^b^	gi|119720766 hydrogen-transporting ATP synthase ( *B. rapa*)_At5g13450	26.8/9.3	21.9/6.4	30.2	14.8	6
139 ^a^			21.8/6.3	136.3	35.6	27
128 ^b^	gi|219902057 unnamed protein product ( *B. napus*)_At5g13120	27.3/9.9	19.2/8.0	50.3	26.2	11
130 ^b^			17.1/6.5	70.3	31.7	14
134 ^a^	gi|75313132 CLP protease proteolytic subunit 6 ( *A. thaliana*)_At1g11750	29.4/9.6	20.0/4.1	20.2	10.7	4
135 ^a^	gi|295421165 unnamed protein product ( *B. napus*)_At3g61440	39.8/8.9	32.0/7.1	70.3	18.2	12
138 ^a^	gi|20140684 translationally-controlled tumor protein homolog_( *B. oleraceae*)At5g61770	19.0/4.5	20.8/5.2	30.2	19.6	6
140 ^a^	gi|27734544 40S ribosomal protein S5-1 ( *A. thaliana*)_At2g37270	23.0/10.1	23.7/8.4	20.3	13.5	4
142 ^a^	gi|296512706 unnamed protein product ( *B. napus*)_At2g29500	17.8/6.4	14.6/6.5	30.2	21.2	6
143 ^a^	gi|126095240 calmodulin ( *N. caerulescens*)_At3g43810	16.8/3.9	20.0/6.6	30.3	31.5	4
148 ^a^	gi|730129 Nucleoside diphosphate kinase 1 ( *A. thaliana*)_At4g09320	16.5/6.4	14.2/7.3	20.2	16.8	3
169 ^b^	gi|257333266 unnamed protein product ( *B. napus*)_At1g27130	24.9/5.9	23.0/4.1	50.3	20.7	7
170 ^b^	gi|157849652 pollen coat protein ( *B. rapa*)_At1g76180	21.6/5.2	22.5/4.1	20.3	17.70	3
178 ^b^	gi|12585530 vacuolar proton-ATPase subunit F ( *A. thaliana*)_At4g02620	14.3/6.1	10.5/6.2	50.2	42.2	9
183 ^b^	gi|297800752 ATARD2 with metal ion binding activities ( *A. lyrata* subsp. *lyrata*)_At4g14710	23.3/4.9	20.8/5.8	70.3	28.1	15

^a^ Protein spots specifically detected at harvest; ^b^ Protein spots specifically detected after cooking; ^c^ Protein spots specifically detected both at harvest and after cooking; ^d^ Theoretical molecular weight and p*I*; ^e^ Experimental molecular weight and p*I*; ^f^ The score defines the sequence identity; ^g^ No. of peptides identified by MS/MS.

**Table 5:** Putative protein allergens in *ipt*-transgenic lines 102 and 103.

Spot No.	Allergen-Scientific Name ^a^	Allergen Description_Species ^b^	Category ^c^	Length/Identity ^d^	Score ^e^	Allergen Quantity (%) ^f^ at Harvest		Allergen Quantity (%) ^f^ after Cooking	
						102	103	102	103
11	Alt a 4	Protein disulfide-isomerase (PDI) (EC 5.3.4.1)_ *Alternaria alternata*	Areo Fungi	327/29%	116	0.30 ± 0.08	0.25 ± 0.01	0.48 ± 0.35	0.31 ± 0.10
28	Hev b 10.0101	Superoxide dismutase [Mn], mitochondrial (EC 1.15.1.1)_ *Hevea brasiliensis*	Contact	230/79%	402	0.07 ± 0.10	0.04 ± 0.06	0.09 ± 0.13	0.03 ± 0.04
41	Mal f 2	Putative peroxiredoxin (1.11.1.15) (Thioredoxin reductase) (MF1)_ *Malassezia furfur*	Contact	121/42%	112	0.25 ± 0.05	0.58 ± 0.09	ND	ND
43				121/41%	109				
44	Bet v 7	Peptidyl-prolyl *cis*–*trans* isomerase (EC 5.2.1.8)_*Betula pendula*	Aero Plant	169/86%	318	ND	ND	0.19 ± 0.02	0.40 ± 0.57
128, 130				171/63%	225				
64	Mala f 4	Malate dehydrogenase (EC 1.1.1.37)_ *M. furfur*	Contact	344/52%	336	ND	0.08 ± 0.03	ND	ND
82, 123	Mal f 3	Putative peroxiredoxin (1.11.1.15) (Thioredoxin reductase) (MF2)_ *M. furfur*	Contact	166/39%	133	ND	0.76 ± 0.47	ND	1.31 ± 0.25
121, 129	Gly m	Kunitz trypsin inhibitor, Glycinin_ *Glycine max*	Food Plant	200/25%	46	ND	4.04 ± 1.14	0.23 ± 0.33	6.48 ± 4.15
122, 173, 184				164/30%	69.3				
126	Car b 1	Major pollen allergen Car b 1 isoform 2 (Allergen Car b I)_ *Carpinus betulus*	Aero Plant	158/34%	74.9	ND	ND	0.38 ± 0.54	ND
143	Tyr p	Torponin C_ *Tyrophagus putrescentia*	Aero Insect	148/43%	113	0.09 ± 0.13	ND	ND	ND

Data are mean ± SD; ^a^ The scientific name of the best-hit allergen provided by the Allergen Database for Food Safety; ^b^ Description of the protein allergen provided by the Allergen Database for Food Safety; ^c^ Category defined in the Allergen Database for Food Safety; ^d^ Length of query compared and protein identity between the query and allergen; ^e^ The score defines the sequence identity; ^f^ Percentage allergen quantity of total amount of proteins; ND, protein not detected.

Unavoidably, most of these putative protein allergens play important physiological functions in plants and help plants in the defense against stress. PDIs, the putative Alt a 4-type allergens, are molecular chaperones that help in the proper formation of disulfide bonds during protein folding [26]. MnSODs, the putative Hev b 10.0101-type allergen predominantly found in mitochondria and peroxisomes, act as antioxidants and protect cellular components against oxidation by reactive oxygen species with biotic and abiotic stresses [27]. Peroxiredoxins, the putative Mal f2- and Mal f3-type allergens, function in the plant defense against oxidative stresses [28]. Besides belonging to Gly m-type allergen, trypsin inhibitors also function as toxins and anti-nutritional substances that protect plants against herbivores by inhibiting the digestive proteases of herbivores [11,29]. However, trypsin inhibitors exist widely in wild edible plant food and can achieve more than 8 µg·mg^−1^ dry weight [30]. Both exogenously supplied and transgenic-increased cytokine levels can increase levels of stress-related proteins [23]. Thus, *ipt*-transgenic broccoli may possess a higher level of these proteins, stress-protection related in plants, but putative allergens for consumers, than non-transgenic controls. Also, these physiologically important proteins may be too low in quantity in control plants to be detected by silver staining.

### 2.4. Level of Glucosinolates Increased in Inbred Broccoli

Because of the anti-cancer and anti-nutritional activities of glucosinolates, we compared their quantities in *ipt*-transgenic lines and controls. The content of glucosinolates was higher in the *ipt*-transgenic line 103 and the non-transgenic control line 104 as compared with the parental GK line, which indicated independence of the transgenic *ipt* gene and glucosinolate content. At harvest, glucosinolate content was lower in GK broccoli than in line 103 and line l04 (38.7 ± 7.3 *vs.* 51.7 ± 2.6 and 51.2 ± 9.3 µmol 100 g^−1^ dry weight [DW], respectively). After broccoli storage at 25 °C for 4 days, the content was reduced more than 35% (25.2 ± 4.8 *vs.* 31.7 ± 4.0 and 26.2 ± 8.2 µmol 100 g^−1^ DW, respectively).

Glucosinolates are heat-labile during processing [31], and their content was decreased during postharvest storage in our research. Different animals exhibit different susceptibility to glucosinolates, ranging from 0.5 µmol·g^−1^ diet for rats to 10 µmol·g^−1^ diet for steers [16]. In humans, clinical evidence showed that uptake of about 300 µmol glucosinolates per day did not have significant adverse effects on human health [32]. Regarding our data, 300 µmol glucosinolates represented about 600 g DW of *ipt*-transgenic broccoli, 5 times higher than the 120 g/day for human uptake, which mimics 0.52 g/day uptake for a mouse.

### 2.5. Mice Fed Ipt-Transgenic Broccoli Showed Normal Phenotype and Physiologic Features

**Figure 3:**
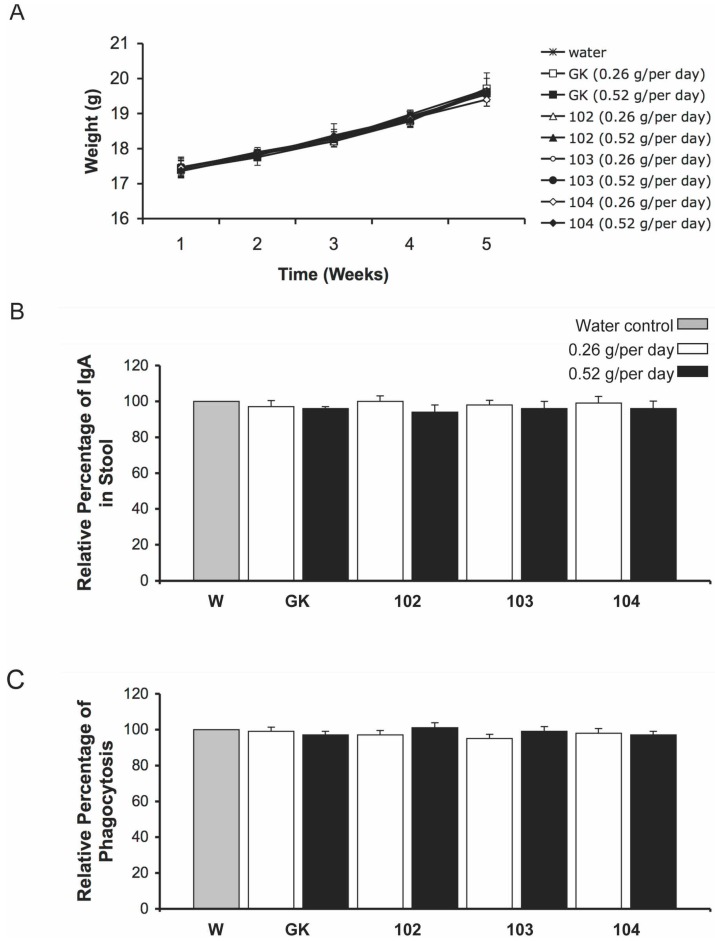
Growth, mucosal immunity and phagocytosis activity of mice fed *ipt*-transgenic broccoli. (**A**) The weight of mice fed 2 levels of broccoli florets, 0.26 and 0.52 g, over time. Data are mean ± SD (*n* = 8); (**B**) Relative IgA level in mouse stools. *x*-axis indicates mice fed water (W) and broccoli of the parental line Green King (GK), *ipt*-transgenic lines 102 and 103, and inbred control line 104. The IgA level of control-water–fed mice (gray column) in stools was set to 100%. Data are mean ± SD of triplicate experiments; (**C**) Phagocytosis activity in control-water–fed mice (gray column) was set to 100%. Data are mean ± SD of triplicate experiments (*n* = 8).

We fed mice *ipt*-transgenic broccoli to evaluate the effects of the increase in potential protein allergens on physiologic features and the immune system. The changes including potential allergic proteins detected in *ipt*-transgenic broccoli and the increased glucosinolate content could suggest adverse effects of *ipt*-transgenic broccoli as food for humans. We fed 8-week-old mice 0.26 and 0.52 g/day of control and *ipt*-transgenic broccoli. The weight of all mice increased gradually during feeding, with no significant difference in weight and morphologic appearance of mice fed fresh *ipt*-transgenic lines 102 and 103 and control broccoli (Figure 3A). The mice fed cooked broccoli showed the same results (data not shown). Therefore, both regimes likely do not have adverse affects on mouse growth.

We assessed mucosal immunity of mice by determining immunoglobulin A (IgA) level in stools at day 28 after broccoli feeding. IgA is the most abundant mucosal antibody and plays an important role in mucosal immunity [33]. It functions to neutralize toxins and pathogenic microbes in high- and low-affinity modes to contain the dense commensal microbiota within the intestinal lumen [34]. Thus, the balance of IgA in the gastrointestinal tract is an important indicator of animal health. IgA levels in stool did not differ for mice fed either levels of *ipt*-transgenic or control broccoli (Figure 3B). Thus, the putative allergens in the *ipt*-transgenic broccoli with both levels did not interfere in IgA homeostasis in the mouse gastrointestinal system.

**Figure 4:**
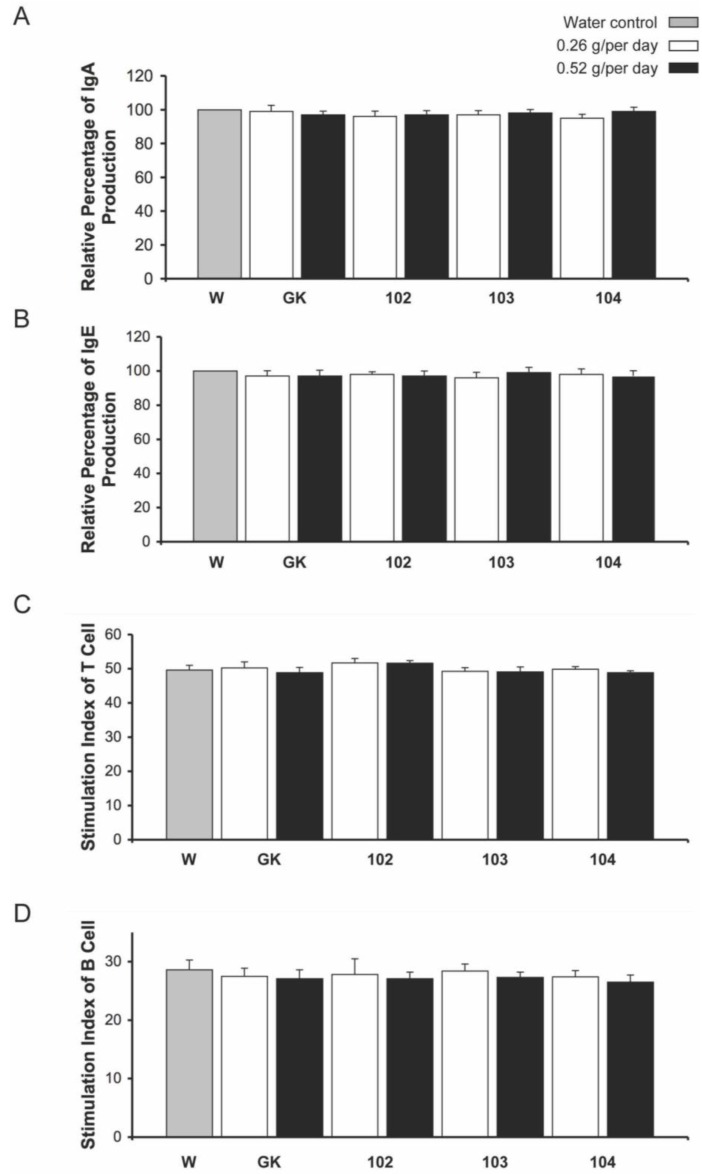
Serum antibody levels of immunoglobulin A (IgA) and IgE, and stimulation of immune cells in mice fed *ipt*-transgenic broccoli. (**A**) IgA level; (**B**) IgE level. Antibody level for control-water–fed mice (gray column) was set to 100%. Data are mean ± SD of triplicate experiments (*n* = 8); Stimulation index for (**C**) T cells and (**D**) B cells. Data are mean ± SD of triplicate experiments (*n* = 8).

After mice were fed *ipt*-transgenic broccoli for 28 days, we determined the effect of the broccoli on the mouse immune system by detecting blood phagocytosis activity, IgA and IgE levels in serum, and splenocyte populations of major immune cells and mitogen-responsive activities of T and B cells. The relative proportion of macrophages with phagocytosis activity did not differ for mice fed either level of *ipt*-transgenic or control broccoli (Figure 3C). Serum levels of IgA and IgE showed the same results (Figure 4A,B). The relative proportion of CD4+ helper and CD8+ cytotxic T cells and CD19+ B cells was similar among all treatments (Table 6), as was the mitogen-caused proliferation index of T and B cells (Figure 4C,D).

In principle, the immune system encounters food allergens in the digestive system. Although IgA is the most abundant antibody in the mucosal immune system and manages the homeostasis of commensal microflora, IgE plays a major role in inducing hypersensitivity [35] for food allergies by recognizing food allergens and eliciting downstream immunoprotective responses, including activation and proliferation of B and T cells; specification of B cells into CD19+ B cells and T cells into CD4+ helper and CD8+ cytotxic T cells; increased phagocytotic activity of macrophages; and production of antibodies. In addition to IgE-mediated food allergy, another T-cell–involved non-IgE-mediated pathway is related to food allergy [36]. Mice fed *ipt*-transgenic broccoli showed no allergen-induced hypersensitivity syndrome, similar to those fed non-transgenic broccoli and water, which suggests a safe uptake level and/or lack of allergic induction with this GM crop.

**Table 6:** Mean percentage of immune cells in mouse spleens after broccoli feeding.

Broccoli Line, Level (g/day)	Immune Cell Population		
	CD3/4	CD3/8	CD19
GK			
0.26	33.48 ± 1.38	21.46 ± 1.63	44.27 ± 2.28
0.52	32.81 ± 1.99	21.51 ± 1.08	44.15 ± 3.22
102			
0.26	32.76 ± 1.26	22.12 ± 1.36	42.12 ± 1.67
0.52	34.86 ± 1.11	21.05 ± 2.88	43.89 ± 2.11
103			
0.26	32.12 ± 1.21	22.42 ± 2.54	44.16 ± 1.66
0.52	32.15 ± 1.85	22.24 ± 2.57	44.26 ± 2.54
104			
0.26	33.26 ± 1.52	21.52 ± 1.55	44.52 ± 1.44
0.52	33.33 ± 1.52	22.16 ± 2.24	43.62 ± 2.27

Data are mean ± SD. GK, Green King; its selfed line 104 and *ipt*-transgenic selfed-T_5_ lines 102 and 103.

## 3. Experimental Section

### 3.1. Plant Materials

We used broccoli (*Brassica* *oleracea* var. *italica* cv. Green King; GK), its fifth generation (T_5_) inbred line 104 and *ipt*-transgenic T_5_-inbred lines 101, 102 and 103, containing the agrobacterial *ipt* gene driven by the senescence-associated *SAG-13* promoter [21]. The transgenic plants were homozygous plants with single T-DNA insertion [21]. The plants were grown in an isolated field at the Taiwan Agricultural Research Institute (TARI), Wufeng, Taichung, Taiwan. The harvested floral heads were transported immediately to the laboratory in a cold-storage cabinet [22]. They were sampled directly at harvest, after postharvest storage at 25 °C in the dark for 4 days, or after being cooked in boiling water for 5 min. The floral heads were separated into small inflorescences/florets and mixed by equal weight from individuals. For proximate analysis including the content of water, crude protein, crude fat, crude fiber, ash, and carbohydrates (CHO), chemical contents including ascorbic acid, titratable acidity, formol-N, free sugar and dissolved solids, and mineral contents, mixed florets were collected from 5 individuals and 3 biological repeats were performed. For protein extraction and analysis, mixed florets were obtained from 3 individuals and stored at −80 °C. For glucosinolate extraction and analysis, the mixed florets were freeze-dried under vacuum, ground into powder with use of a blender and stored at −80 °C. For the mouse-feeding experiment, mixed florets were homogenized into slurry and stored at −20 °C.

### 3.2. Proximate Analysis of Macronutrients, Chemicals and Minerals

Fresh sample or dry ground sample were used for analyses. Dry ground samples were prepared by grinding into powder after drying at 60 °C for 18 h. Water content was determined by oven method [37]. Fresh sample was dried in an oven at 105 °C for at least 18 h. The loss in weight was calculated and represented the water content of the samples. Crude protein content was determined by a microdiffusion method of Kjeldahl digest [38]. Briefly, 200-mg dry samples were digested with concentrated H_2_SO_4_ by the micro-Kjeldahl method [37]. After volumetric dilution, the Kjeldahl digest was mixed with 10N NaOH in a microdiffusion plate. The liberated ammonia was trapped in 4% boric acid solution and titrated with 0.01N HCl to determine nitrogen content. Crude protein was calculated by multiplying nitrogen content with a factor of 6.25. For crude fat content, 1-g dry ground samples were extracted with ether for 1 h in a Tecator Soxtec System (Model HT2 1043, Foss, Hoganas, Sweden) [37]. Crude fiber was determined by modified fritted glass crucible method [37]. Defatted sample obtained from fat extraction was digested successively by near-boiling 1.25% H_2_SO_4_ and 1.25% NaOH by use of a Tecator Fibertec System (Model 1020, Foss, Hoganas, Sweden). Ash content was measured by subtraction of acid-insoluble ash from total ash. Total ash content was determined by incineration of 5-g fresh samples in a muffle furnace at 550 °C for 8 h [37]. The ash from total ash determination was then boiled with 3N HCl, filtered through an ashless filter paper and washed with 0.2N HCl. All the filtrate was collected for further mineral determination. The acid-insoluble residue with the ashless filter paper was incinerated at 550 °C to measure acid-insoluble ash. The CHO content was determined by the sample weight minus the sum of water, crude protein, fat, fiber and ash. Mineral composition was determined by analyzing the acid-soluble filtrate from ash content determination with an inductively coupled plasma-atomic emission spectrophotometer (ICP-AES, JY 38 Type III, HORIBA Jobin-Yvon, Longjumeau, France).

Free sugar was determined by reflux extraction of 100-mg dried samples with 5 mL hot 80% ethanol for 30 min 4 times. All the extract was mixed and filtrated through a glass fiber filter. The filtrate was analyzed for sugar content (glucose equivalent) by a phenol–sulfuric-acid method [39]. The content of dissolved solids was determined by a modified AOAC method [37]. Slurry fresh samples (10 grams, broccoli:water = 1:2) were reflux-extracted with 200 mL boiling water for 30 min and filtrated through a Whatman #41 filter paper (pre-dried at 105 °C and pre-weighed) in a Buchner funnel. The filtrate was collected for further determination of titratable acidity and formol-N content. The solid residue on the filter paper was washed with hot distilled water and dried at 105 °C for calculating the dry weight of dissolved solids. The filtrate from the determination of dissolved solids was analyzed for titratable acidity by an alkaline titration method and formol-N content by a formol titration method [37]. The filtrate (100 mL) was titrated to pH 8.1 with 0.1N NaOH to calculate titratable acidity. The titrated filtrate was mixed with 2 mL of 37% formol solution, pH 8.1, and then titrated back to pH 8.1 with 0.01 N NaOH to determine formol-N content. Ascorbic acid was determined by an indophenol titration method [37]. Fresh samples were extracted with HPO_3_–HOAc–H_2_SO_4_ solution and titrated with 2,6-dichloroindophenol standard solution.

### 3.3. Protein Extraction, Electrophoresis, Gel Staining and Image Analysis, and Identification by Liquid Chromatography-Electrospray Ionization-Tandem Mass Spectrometry (LC–ESI-MS/MS)

The methods of protein extraction and electrophoresis to LC–ESI-MS/MS were as in our previous study [22], except the 2DE gels were silver stained. In brief, 1 g mixed florets was ground into powder in liquid nitrogen with use of a mortar and pestle. Protein was extracted with 2 mL acetone containing 10% trichloroacetic acid and 0.3% DTT. The concentration of protein was detected by the Bio-Rad protein assay with bovine serum albumin as the standard. 2-DE was performed twice for each sample starting with 240 µg total protein by use of an Immobilin DryStrip, pH 3–10 NL, 13 cm (GE Healthcare, Uppsala, Sweden), on a Multiphor II (Amersham, Taipei, Taiwan) for the first dimension and 12.5% SDS-PAGE for the second dimension [22]. The gels were silver-stained with use of the PlusOne Silver Staining Kit (GE Healthcare). Gels were scanned by use of an image scanner (Amersham). Protein spots on the gel images were detected and matched by use of ImageMaster 5.0 (Amersham, Uppsala, Sweden) [22] after manual correction of spots and rematching. The protein quantity of a spot was determined by the volume of the spot, and the spot volume was normalized to that of total protein on each gel and presented as a percentage (%). To identify the differential expressed proteins that may be harmful to human health in *ipt*-transgenic florets, proteins exclusively detected in florets of *ipt*-transgenic broccoli were selected for in-gel digestion and LC–ESI-MS/MS (Thermo Scientific, Waltham, MA, USA) by the Proteomics Core Lab at the Institute of Plant and Microbial Biology, Academia Sinica, Taipei, Taiwan [22]. The acquired MS/MS data were used for sequence identification with use of Mascot v2.3 (Matrix Sciences, Boston, MA, USA) with the *Brassica* Database (347,992 protein entries) from UniProtKB/Swiss-Prot, with significance threshold *p* = 0.05 to obtain the best-hit protein candidates. The matched proteins were accepted only when they had at least 3 MS/MS peptide hits. We also considered the experimental and theoretical p*I*, molecular weight, protein score and sequence coverage of candidate proteins.

### 3.4. Search for Functional Homologs and Potential Allergenic Property

The best-hit protein sequences identified by LC–ESI-MS/MS underwent a BLAST search in the National Center for Biotechnology Information database [40] for functional homologs and the Allergen Database for Food Safety [41] for potential allergens.

### 3.5. Glucosinolate Extraction and Analysis

To extract glucosinolates, 4 mL boiled de-ionized water (ddH_2_O) was added to 0.2 g broccoli powder and shaken under 150 rpm for 30 min. After centrifugation, a 250-µL supernatant aliquot was diluted with 2750 µL ddH_2_O and passed through a DEAE Sephadex A25 column (GE Healthcare) pre-treated with ddH_2_O [42]. Glucosinolates were eluted with 3 mL of 0.1 M phosphate buffer, pH 6.6, into a test tube containing 500 µL myrosinase, an enzyme that hydrolyzes glucosinolates into isothiocyanates and releases glucose [43], and incubated at 37 °C overnight. A series-diluted sinigrin was used as an external standard. An amount of 1 ml of the reaction solution was mixed with an equal volume of Glucose Assay Reagent (Sigma, St. Louis, MO, USA) and incubated at 30 °C for 15 min [44]. The absorbance of glucose at 340 nm was detected by use of a Beckman Coulter DU 800 UV/Visible Spectrophotometer. Standard curves for glucose and sinigrin were generated for normalizing the concentration of glucosinolates in broccoli on a dry weight (DW) basis.

### 3.6. Mice

Balb/c mice (8 weeks old) were from the National Laboratory Animal Center (Taipei, Taiwan) and were maintained in the Animal Center of China Medical University. The animal room was kept at a 12-h light/dark cycle with constant temperature and humidity. All procedures followed the Guide for the Care and Use of Laboratory Animals and were approved by the animal care committee at China Medical University, Taichung, Taiwan (identification code: 96-16-N, 1 June 2007).

### 3.7. Broccoli Feeding of Mice

Mice were randomly divided into 9 groups (*n* = 8 mice each) for treatment: feeding with an equal volume of water (control) or 0.26 and 0.52 g florets of line GK, line 104, or *ipt*-transgenic lines 102 and 103 per day, which mimics 60 and 120 g broccoli uptake per day, respectively, for a 60-kg human adult [45]. Animals had free access to the food.

### 3.8. Determination of Immunoglobulin (Ig) Level in Stool and Serum

We collected stools from each group at day 28. The IgA in stool was extracted as follows. Feces were obtained at the same times and resuspended in phosphate buffered saline (PBS) plus 1% fetal calf serum (Life Technologies, Paisley, Scotland) supplemented with pepstatin (1:1000; Fluka, Buchs, Switzerland) at 0.1 mg/mL. Samples were mechanically disaggregated and vortexed for 2 min, followed by 2 centrifugations at 4 °C for 20 min at 14,000 rpm, and the supernatant was stored at −70 °C [46]. Blood samples were taken from the retro-orbital venous plexus of mice after 28-day treatment, and separated by centrifugation. Plasma was stored at −70 °C. The levels of IgA and IgE were determined by ELISA (Sigma, Poole, UK).

### 3.9. Quantification of Phagocyte Activity in Mice

Quantification of phagocyte activity involved the Phagotest kit (ORPEGEN Pharma, Heidelberg, Germany) [47]. Briefly, 100 mL heparinized whole blood was incubated with a half-volume of fluorescein isothiocyanate (FITC)-labelled *Escherichia coli* at 37 °C; a negative control sample remained on ice. Phagocytosis was stopped by placing the sample on ice and adding solution to quench the FITC fluorescence of surface-bound bacteria, leaving the fluorescence of internalized particles unaltered. After a wash, phagocyte activity was determined by counting the number of ingested bacteria by FACscan flow cytometry (Becton Dickinson, San Jose, CA, USA). Data analysis involved Cell Quest Software (Becton Dickinson), and phagocyte activity was calculated by subtracting the proportion of phagocytes that ingested FITC-labeled *E. coli* at 37 °C from that of the ice control.

### 3.10. Determination of Cell Populations in Splenocytes

After 28-day treatment, mice were killed and spleens were crushed into a single-cell suspension to obtain splenocytes as described [48]. For surface staining, 1 × 10^6^ splenocytes were incubated with FITC-conjugated anti-CD3 (Leinco Technologies, St. Louis, MO, USA) or phycoerythrin [PE]-conjugated anti-CD4, PE–anti-CD8, or PE–anti-CD19 antibodies (PharMingen, San Diego, CA, USA) at 4 °C for 30 min [49]. Cells were washed twice, suspended in 0.5 ml PBS and underwent FACScan analysis. A total of 10,000 cells were counted and the frequency of each cell surface marker was determined by use of associated software. Flow cytometry was regularly calibrated with CaliBRITE beads (Becton Dickinson).

### 3.11. Assay of Proliferation of B and T Cells

Proliferation of B and T cells was measured by [3H]-thymidine incorporation assay [50]. Briefly, 2 × 10^5^ splenocytes were co-cultured with 10 µg/mL concanavalin A (Con A; Sigma, MO, USA) to stimulate T-cell proliferation or lipopolysaccharide (LPS; Sigma, MO, USA) to stimulate B-cell proliferation for 72 h. During the last 18 h, cells were pulsed with 1 µCi [3H]-thymidine (Amersham France SA, Les Ulis, France). The proliferative response was expressed as stimulation index: (count per minute (CPM value) of experiment)/(CPM value of control) × 100%.

### 3.12. Statistical Analysis

All data are expressed as mean ± SD. Statistical analysis involved one-way ANOVA followed by Dunnett’s *post-hoc* test, and significant difference was set at *p* < 0.05.

## 4. Conclusions

Our results show a general comparable balance in nutritional, chemical and mineral constituents between *ipt*-transgenic broccoli and their controls. We found altered levels of magnesium and carbohydrates and some differentially expressed proteins with homology to protein allergens. However, these proteins have important physiological and functional roles against plant stresses. Their putative roles as allergens still need further study. In our research, the compositional and proteomic changes do not reach a threshold to affect growth or induce an immune response in mice under normal broccoli feeding.

## Supplementary Materials

Supplementary materials can be found at http://www.mdpi.com/1422-0067/15/9/15188/s1.

## Supplementary Files

Supplementary File 1
