# Supplementary material for: Compositional and Proteomic Analyses of Genetically Modified Broccoli (Brassica oleracea var. italica) Harboring an Agrobacterial Gene
Source: Int J Mol Sci. 2014 Aug 28;15(9):15188–209. doi: 10.3390/ijms150915188 (PMC4200750; doi:10.3390/ijms150915188)
Supplement: Supplementary File 1 [file ijms-15-15188-s001.pdf]

Supplemental Information

Figure S1. 2D gel electrophoresis of *ipt*-transgenic broccoli at harvesting and after cooking. Protein composition of *ipt*-transgenic broccoli lines 102 and 103 and non-transgenic control were compared at harvesting and after cooking. The relative molecular weight of protein is indicated at the left and the pH value at the bottom of each gel. The *ipt* transgenic broccoli-specific protein spots are marked with arrows and an Arabic number.

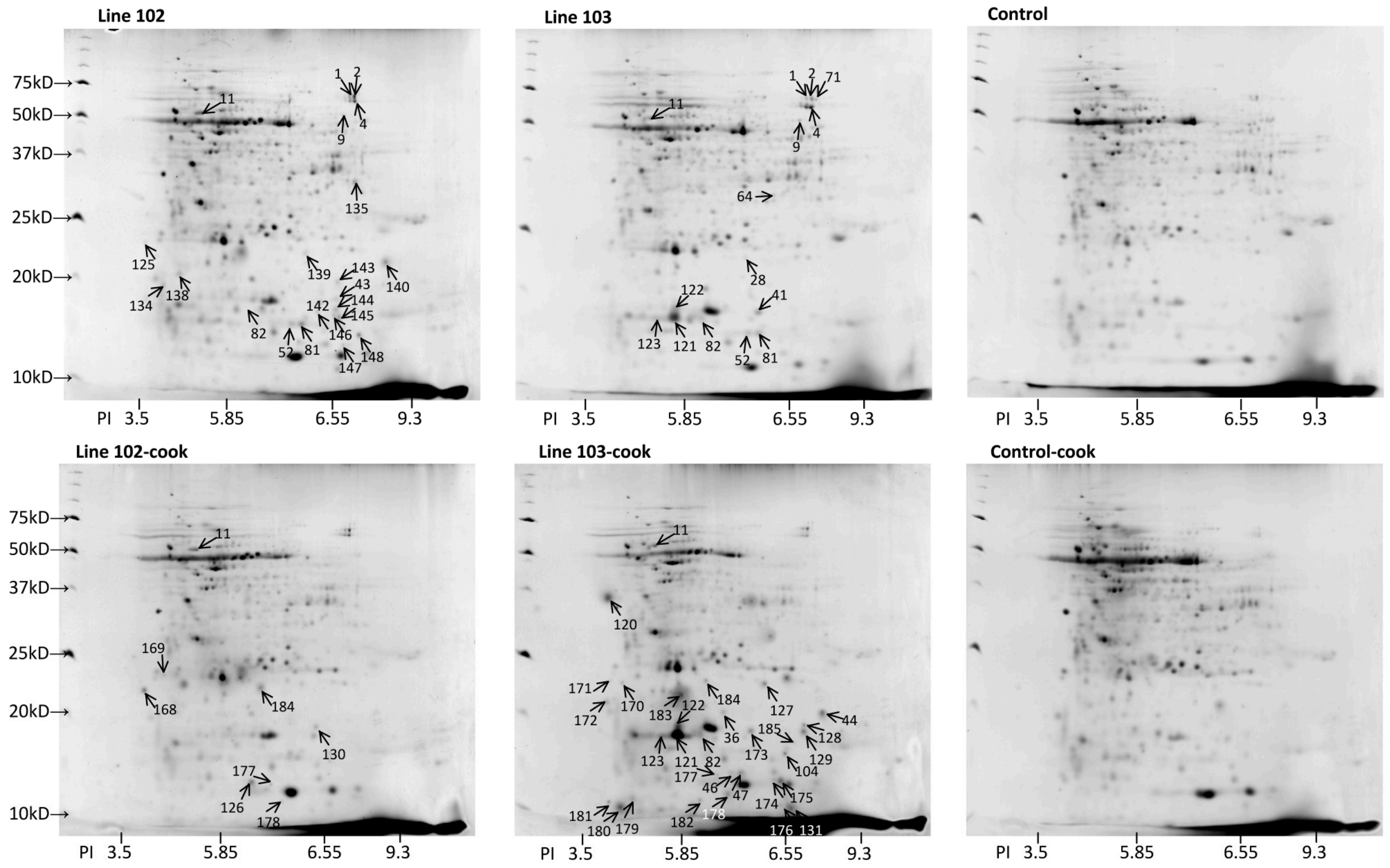

Table S1. MS/MS match peptide of proteins specifically detected in *ipt*-transgenic broccoli.

Spot No.	Peptide Matched Number	Dupes	Peptide Sequence	Peptide Score	Peptide Charge
1	18		K.IEAIAK.S	4	2
			K.FMNIK.C	9	2
			K.RSFSDIMFR.R	7	2
		1	K.TQYSFSHDASK.	47	2
			R.LDIDPASITWR.R	10	2
		2	K.SYGASGVEYSDQAEK.Q	91	2
		1	R.YSGLTPQCAIVVATVR.A	86	2
			K.STTTVGLCQALGAYLDK.K	25	3
			K.STTTVGLCQALGAYLDKK.V	33	3
		1	K.AGDPITADDLGVGGALTVLMK.D	5	3
		2	K.LVGPGGFVVTEAGFGSDIGTEK.F	136	2
4	5		K.ACQNITQPLR.F	2.96	2
			K.STTTVGLCQALGAYLDKK.V	2.55	3
		1	K.SYGASGVEYSDQAEK.Q	4.94	2
			R.SFSDIM*FR.R	2.28	2
2	13		R.ELELSR.R	19	2
			R.AVEALNGKK.F	12	2
			K.VSEALDVLR.N	11	2
		2	R.RSGVGNLFVK.N	13	2
			K.VMRDPSGTSK.G	4	3
			R.RYEQGSSDGGNK.F	16	3
			K.LNYSYLNGKMIR.I	6	3
		1	R.ELFAEFGTITSCK.V	82	2
		1	R.GYGFVQFDTEDSAK.N	44	2

Table S1. *Cont.*

Spot No.	Peptide Matched Number	Dupes	Peptide Sequence	Peptide Score	Peptide Charge
9	50	1	-.ILGVHIM*APNAGELIHEAVLAINYDASSEDIAR.-	6.54	3
			-.LTVEPAEGGEQSILEADVVLVSAGR.-	2.56	2
			-.VCHAHPTMSEALK.-	2.47	2
			K.AEEDGVACVEFIAGK.H	4.14	2
			K.AIDNAEGLVK.I	3.52	2
		2	K.ALLHSSHMYHEAK.H	4.10	2
			K.EAAM*ATYDKPIHI.-	3.10	2
		1	K.EAAMATYDKPIHI.-	3.29	2
			K.FPFMANSR.A	2.42	2
		2	K.GKHIIVATGSDVK.S	4.24	2
		1	K.HGHVDYDKVPGVVYTHPEVASVGK.T	7.63	3
		2	K.HIIVATGSDVK.S	3.47	2
		1	K.IVSSTGALSLSEVPK.K	3.83	2
		1	K.IVSSTGALSLSEVPKK.L	4.17	2
		1	K.KIVSSTGALSLSEVPK.K	5.52	2
			K.KIVSSTGALSLSEVPKK.L	4.64	3
		1	K.KIVSSTGALSLSEVPKK.L	5.22	2
			K.KLIVIGAGYIGLEM*GSVWGR.L	5.60	2
			K.KLIVIGAGYIGLEMGSVWGR.L	2.97	3
		2	K.LIVIGAGYIGLEM*GSVWGR.L	6.85	2
		1	K.LIVIGAGYIGLEM*GSVWGR.L	5.67	3
		1	K.SLPGITIDEKK.I	2.70	2
		1	K.VPGVVYTHPEVASVGK.T	4.77	2
		1	K.VSSVEVDLPAMLAQK.D	3.98	2
			R.FLSNVPGVYAIGDVIPGPMLAHK.A	2.37	2
			R.GALGGTCLNVGCIPSK.A	4.70	2
		1	R.TPFTSGLDLEK.I	3.19	2
			R.VGKFPFM*ANSR.A	2.59	3
		1	R.VGKFPFMANSR.A	2.37	2

Table S1. *Cont.*

Spot No.	Peptide Matched Number	Dupes	Peptide Sequence	Peptide Score	Peptide Charge
11	74		-.DPSNHPYVSK.-	2.45	2
			-.KQSGPASAEIK.-	2.91	2
		4	K.AAAELSSQSPPIFLAK.I	4.51	2
			K.ANVVVDQIESWM*K.D	4.32	2
			K.ANVVVDQIESWM*KDFKDGK.V	3.18	3
		1	K.ANVVVDQIESWMK.D	4.67	2
			K.DPSNHPYVSK.F	2.13	2
			K.ESSIPLVTVFDKDPSNHPYVSK.F	3.10	3
			K.FVKESSIPLVTVFDKDPSNHPYVSK.F	4.79	3
		1	K.GQDLAFLVGDAESSQGALQYFGLEESQVPLIIIQTSDSKK.Y	6.39	3
		1	K.IDASEESNKGIANEYK.I	4.53	2
		1	K.IDASEESNKGIANEYK.I	4.33	3
		1	K.IDASEESNKGIANEYKIQGFPTIK.I	6.38	3
		1	K.KQSGPASAEIKSADGAAEVIGEK.S	4.44	3
		1	K.LAPILDEVALAFQNDPSVIVAK.L	4.98	3
		1	K.LAPILDEVALAFQNDPSVIVAK.L	5.39	2
			K.LDATANDIPSDTFDVK.G	5.38	2
		1	K.LRADYDFAHTLDAK.F	4.15	2
		5	K.LSGEEFDSFM*AVAEK.L	5.05	3
		2	K.LSGEEFDSFMAVAEK.L	5.90	3
			K.NVLIEFYAPWCGHCQK.L	5.13	2
			K.QSGPASAEIKSADGAAEVIGEK.S	3.81	3
			K.SADGAAEVIGEK.S	2.82	2
			K.SADGAAEVIGEK.S	2.24	2
			K.SIQDYNGPR.E	2.05	2
		4	K.SIQDYNGPR.E	2.12	2
		2	K.SQPIPAENNEPVK.V	3.20	2
			K.SQPIPAENNEPVKVVVAESLDDM*VFNSGK.N	5.20	3

Table S1. *Cont.*

Spot No.	Peptide Matched Number	Dupes	Peptide Sequence	Peptide Score	Peptide Charge
		1	K.SQPIPAENNEPVKVVVAESLDDMVFNSGK.N	4.73	3
			K.SVVAVGVFPK.L	2.84	2
		3	K.VVVAESLDDM*VFNSGK.N	5.33	2
		3	K.VVVAESLDDMVFNSGK.N	4.78	2
		1	K.VVVYEGSR.T	2.31	2
			R.LFKPFDELFVDSKDFNGEALEK.F	3.59	3
		1	R.SADGKVVVYEGSR.T	4.01	3
			R.SGEHKTEESAAKDEL.-	3.78	3
			R.SGEHKTEESAAKDEL.-	3.01	2
		1	R.TKEDFISFIEK.N	4.01	2
28	8	1	-.YASEVYEK.-	2.17	2
			K.FNGGGHVNHSIFWK.N	2.59	2
			K.HHQTYVTNYNNALEQLDQAVNK.G	4.39	3
			K.KLVVDTTANQDPLVTK.G	5.24	2
		2	K.LVVDTTANQDPLVTK.G	4.43	2
36	1		K.NPDQVTENDFAFTGLGK.A	4.8	2
41	14	1	-.SWDEGVSSK.-	2.56	2
			-.VVIFGLPGAYTGVCSQQHVPSYK.-	2.58	3
		1	K.AVNVEEAPSDFK.V	3.41	2
			K.DLSAALLGPR.S	3.15	2
		1	K.SLGLDKDLSAALLGPR.S	4.91	2
			K.VTGAEVILGQI.-	3.11	2
		1	K.VVIFGLPGAYTGVCSQQHVPSYK.S	4.19	2
		2	R.WSAYVEDGKVK.A	3.24	2

Table S1. *Cont.*

Spot No.	Peptide Matched Number	Dupes	Peptide Sequence	Peptide Score	Peptide Charge
43	16		-.SLGLDKDLSAALLGPR.-	3.59	3
		1	K.AVNVEEAPSDFK.V	3.94	2
		1	K.AVNVEEAPSDFK.V	3.20	1
		1	K.DLSAALLGPR.S	3.43	2
		1	K.LSEGTDITSAAPGVSLQK.A	5.48	2
		1	K.SLGLDKDLSAALLGPR.S	5.19	2
			K.VTGAEVILGQI.-	2.82	2
		1	R.WSAYVEDGK.V	2.67	1
		1	R.WSAYVEDGKVK.A	3.45	2
44	12	1	K.FADENFER.K	2.81	2
		1	K.HVVFGQVVEGLDVVK.A	4.49	3
		2	K.IVM*ELYTDKTPK.T	3.23	2
		1	K.IVMELYTDKTPK.T	3.27	2
			K.TDWLDGKHVVFGQVVEGLDVVK.A	2.64	3
		1	R.IVMELYTDK.T	3.15	2
46	14		-.TEYPNAFIR.-	2.12	2
			K.FETLSYLPDLTEVELGK.E	3.24	2
			K.KFETLSYLPDLTEVELGK.E	4.12	3
		1	K.KFETLSYLPDLTEVELGK.E	5.87	2
		1	K.KKFETLSYLPDLTEVELGK.E	4.59	3
			K.KKFETLSYLPDLTEVELGK.E	3.32	2
		1	K.LPLFGCTDSAQVLK.E	4.85	2
			K.TEYPNAFIR.I	2.57	2
			R.IIGFDNNR.Q	2.04	2
		1	R.QVQCISFIAYKPPSFTGA.-	3.74	2

Table S1. *Cont.*

Spot No.	Peptide Matched Number	Dupes	Peptide Sequence	Peptide Score	Peptide Charge
47	8		K.KFETLSYLPDLTEVELGK.E	3.32	3
			K.KKFETLSYLPDLTEVELGK.E	4.12	3
		1	K.LPLFGCTDSAQVLK.E	4.74	2
			K.TEYPNAFIR.I	2.19	2
			R.EHGSTPGYYDGR.Y	2.97	3
		1	R.QVQCISFIAYKPPSFTGA.-	4.73	2
131	3		R.IIGFDNNR.Q	2.51	2
			K.LPLFGCTDSAQVLK.E	4.57	2
			R.QVQCISFIAYKPPSFTGA.-	3.08	2
147	13		K.KFETLSYLPDLTEVELGK.E	4.7	2
			K.KFETLSYLPDLTEVELGK.E	3.7	3
		1	K.KKFETLSYLPDLTEVELGK.E	4.4	3
		1	K.LPLFGCTDSAQVLK.E	4.9	2
			K.TEYPNAFIR.I	2.3	2
		1	R.EHGSTPGYYDGR.Y	3	2
		1	R.IIGFDNNR.Q	2.6	2
		1	R.QVQCISFIAYKPPSFTGA.-	4.6	2
174	10		-.EVDYLLR.-	2.23	1
			-.TEYPNAFIR.-	1.85	1
			K.EVQECKTEYPNAFIR.I	3.36	2
			K.KKFETLSYLPDLTEVELGK.E	4.33	3
		1	K.LPLFGCTDSAQVLK.E	4.76	2
		1	R.EHGSTPGYYDGR.Y	3.09	2
		1	R.QVQCISFIAYKPPSFTGA.-	4.52	2
175	9		-.EVDYLLR.-	2.2	1
		1	K.EVQECKTEYPNAFIR.I	3.5	2

Table S1. *Cont.*

Spot No.	Peptide Matched Number	Dupes	Peptide Sequence	Peptide Score	Peptide Charge
		1	K.LPLFGCTDSAQVLK.E	4.9	2
			R.EHGSTPGYYDGR.Y	3.4	2
			R.IIGFDNNR.Q	2.4	2
		1	R.QVQCISFIAYKPPSFTGA.-	4.5	2
177	14		-.KKFETLSYLPDLTEVELGK.-	4.1	3
			K.FETLSYLPDLTEVELGK.E	4.3	2
		1	K.KFETLSYLPDLTEVELGK.E	4.2	3
		1	K.KFETLSYLPDLTEVELGK.E	5.7	2
			K.KKFETLSYLPDLTEVELGK.E	4.3	3
		1	K.LPLFGCTDSAQVLK.E	2.9	2
		1	K.TEYPNAFIR.I	2.5	2
			R.EHGSTPGYYDGR.Y	2.7	2
		1	R.QVQCISFIAYKPPSFTGA.-	4.4	2
52	14	1	-.M*IMGSVSNHVVNNVACPVTVVK.-	6.66	3
		1	K.ICEAAEHIPLSSLVIGNR.G	5.04	2
		1	K.YGVKPDAETLDIANTAAR.Q	4.86	2
		1	R.IGVAVDFSECSKK.A	3.23	2
		1	R.M*IM*GSVSNHVVNNVACPVTVVK.A	5.01	3
			R.M*IMGSVSNHVVNNVACPVTVVK.A	3.93	3
		1	R.MIM*GSVSNHVVNNVACPVTVVK.A	6.66	3
			R.RIGVAVDFSECSK.K	3.52	3
64	73		-.DDLFNINAGIVK.-	0.14	2
		2	-.SQVVGYM*GDDNLAK.-	0.45	2
		1	-.SQVVGYMGDDNLAK.-	0.57	2
		1	-.VAILGAAGGIGQPLALLM*K.-	0.23	2
		3	K.ALEGADLVIIPAGVPR.K	0.50	1
		10	K.ALEGADLVIIPAGVPR.K	0.46	2

Table S1. *Cont.*

Spot No.	Peptide Matched Number	Dupes	Peptide Sequence	Peptide Score	Peptide Charge
			K.ALEGADLVIIPAGVPR.K	0.33	3
		3	K.GLNGVPDVVECSYVQSTITELPFFASK.V	0.56	2
			K.KLFGVTTLDVVR.A	0.32	1
		3	K.KLFGVTTLDVVR.A	0.42	2
		1	K.LFGVTTLDVVR.A	0.30	2
		1	K.LNPLVSSLSLYDIANTPGVAADVGHINTR.S	0.60	2
		4	K.LNPLVSSLSLYDIANTPGVAADVGHINTR.S	0.68	3
		3	K.NGVEEVLDLGPLSDFEK.E	0.56	2
		4	K.VAILGAAGGIGQPLALLM*K.L	0.57	2
		1	K.VAILGAAGGIGQPLALLMK.L	0.56	2
		5	K.YCPHALVNM*ISNPVNSTVPIAAEIFK.K	0.62	3
		2	K.YCPHALVNM*ISNPVNSTVPIAAEIFKK.A	0.37	3
			K.YCPHALVNMISNPVNSTVPIAAEIFK.K	0.54	3
			K.YCPHALVNMISNPVNSTVPIAAEIFKK.A	0.57	3
		5	R.DDLFNINAGIVK.N	0.28	2
		1	R.KVAILGAAGGIGQPLALLM*K.L	0.36	3
			R.KVAILGAAGGIGQPLALLMK.L	0.41	3
71	13		K.KLPFDR.H	14	2
			R.AFDKIFK.E	12	2
			M.ESLIGLVNR.I	12	2
		1	R.LIEGALGYFR.G	63	2
		1	R.IYGVFDNQLPAALK.K	75	2
			K.DVDPTGERTFGVLTK.L	5	3
		1	R.IASNVSAYVN<u>M</u>VSDTLR.N	7	3
			R.LVDMESAYLTAEFFRK.L	11	3
		1	K.IVSEADGYQPHLIAPEQGYR.R	65	3

Table S1. *Cont.*

Spot No.	Peptide Matched Number	Dupes	Peptide Sequence	Peptide Score	Peptide Charge
81	12		-.MIM*GSVSNHVVNNVACPVTVVK.-	4.58	3
		1	K.ICEAAEHIPLSSLVIGNR.G	4.72	2
		1	K.KYGVKPDAETLDIANTAAR.Q	5.28	3
		1	K.YGVKPDAETLDIANTAAR.Q	4.71	2
		1	R.IGVAVDFSECSK.K	4.64	2
			R.M*IM*GSVSNHVVNNVACPVTVVK.A	5.26	3
			R.M*IMGSVSNHVVNNVACPVTVVK.A	4.52	3
			R.MIMGSVSNHVVNNVACPVTVVK.A	4.68	3
82	24		-.FALLIDNLK.-	2.93	2
			-.LLGLELDLKDK.-	2.61	3
			K.FVADGSGEYTK.L	2.42	2
		1	K.FVADGSGEYTK.L	3.00	2
		1	K.GVDEIICFSVNDPFVM*K.A	5.29	2
		1	K.GVDEIICFSVNDPFVMK.A	5.43	2
		1	K.KVILFGVPGAFTPTCSMK.H	3.42	2
		1	K.LLGLELDLK.D	3.33	2
		1	K.LLGLELDLKDK.G	3.75	2
		1	K.VILFGVPGAFTPTCSM*K.H	4.02	2
		1	K.VILFGVPGAFTPTCSMK.H	4.48	2
		2	K.VTVANVESGGEFTVSSADDILK.A	6.96	2
		1	R.FALLIDNLK.V	2.76	2
123	20	1	-.FALLIDNLK.-	2.7	2
			-.VILFGVPGAFTPTCSM*KHVPGFIEK.-	3	3
		2	K.FVADGSGEYTK.L	3.4	2
			K.GVDEIICFSVNDPFVM*K.A	5.1	2
			K.GVDEIICFSVNDPFVMK.A	4.7	2
		1	K.KVILFGVPGAFTPTCSMKHVPGFIEK.A	4.5	3
		1	K.LLGLELDLK.D	3.2	2

Table S1. *Cont.*

Spot No.	Peptide Matched Number	Dupes	Peptide Sequence	Peptide Score	Peptide Charge
			K.VILFGVPGAFTPTCSM*K.H	3.8	2
		2	K.VILFGVPGAFTPTCSMK.H	4.4	2
			K.VILFGVPGAFTPTCSMKHVPGFIEK.A	2.9	3
		1	K.VTVANVESGGEFTVSSADDILK.A	6.6	2
			R.FALLIDNLK.V	2.6	1
104	14	1	-.GM*IGMVSIGDVVR.-	3.38	2
			-.VGDIM*TEENKLITVTPETK.-	3.18	2
		1	K.DKGM*IGM*VSIGDVVR.A	3.90	2
			K.DKGM*IGMVSIGDVVR.A	3.90	3
		1	K.GMIGM*VSIGDVVR.A	4.25	2
		1	K.VGDIM*TEENKLITVTPETK.V	5.56	3
		1	K.VGDIMTEENK.L	3.33	2
		1	K.VGDIMTEENKLITVTPETK.V	5.84	3
144	9	1	K.DKGM*IGM*VSIGDVVR.A	3.89	2
		1	K.GM*IGM*VSIGDVVR.A	4.40	2
		1	K.VGDIM*TEENKLITVTPETK.V	5.17	3
		1	K.VGDIM*TEENKLITVTPETK.V	4.06	2
			R.AM*QLM*TDNR.I	2.98	2
145	26		-.DKGM*IGMVSIGDVVR.-	3.09	3
		1	-.DKGMIGM*VSIGDVVR.-	3.07	2
			-.GM*IGMVSIGDVVR.-	3.09	2
			-.LITVTPETK.-	2.39	2
			K.DKGM*IGM*VSIGDVVR.A	3.82	2
		1	K.DKGM*IGM*VSIGDVVR.A	3.96	3
		1	K.DKGM*IGMVSIGDVVR.A	4.30	2
		1	K.GM*IGM*VSIGDVVR.A	4.88	2
		1	K.GMIGM*VSIGDVVR.A	4.04	2

Table S1. *Cont.*

Spot No.	Peptide Matched Number	Dupes	Peptide Sequence	Peptide Score	Peptide Charge
		1	K.LITVTPETK.V	2.32	1
			K.VGDIM*TEENK.L	2.88	2
			K.VGDIM*TEENKLITVTPETK.V	5.02	3
		1	K.VGDIM*TEENKLITVTPETK.V	4.13	2
		1	K.VGDIMTEENK.L	3.35	2
		1	R.LNAFIQGGY.-	2.22	1
		1	R.LNAFIQGGY.-	2.84	2
146	24	1	-.GM*IGMVSIGDVVR.-	3.14	2
		1	K.DKGM*IGM*VSIGDVVR.A	4.32	2
		1	K.GM*IGM*VSIGDVVR.A	4.51	2
		1	K.GMIGM*VSIGDVVR.A	3.95	2
		1	K.GMIGMVSIGDVVR.A	4.22	2
		1	K.LITVTPETK.V	2.37	1
		1	K.VGDIM*TEENK.L	3.71	2
		1	K.VGDIM*TEENKLITVTPETK.V	4.06	2
		1	K.VGDIMTEENK.L	3.51	2
		1	R.LNAFIQGGY.-	2.58	2
		3	R.LNAFIQGGY.-	2.67	1
185	5		K.DKGM*IGM*VSIGDVVR.A	3.41	2
		1	K.GM*IGM*VSIGDVVR.A	4.35	2
			K.VGDIM*TEENKLITVTPETK.V	4.85	3
			R.LNAFIQGGY.-	2.45	1
120	35		-.DELDIVIPTIR.-	3.14	2
			-.DLIGPAM*YFGLMGDGQPIGR.-	2.94	2
		1	-.GTLFPM*CGMNLAFDR.-	3.83	2
			-.LSSLDPYFDK.-	3.00	2
			-.YVDAVM*TIPK.-	3.79	2

Table S1. *Cont.*

Spot No.	Peptide Matched Number	Dupes	Peptide Sequence	Peptide Score	Peptide Charge
			K.ASCISFK.D	2.26	2
			K.AVNALEQHIK.N	2.79	2
			K.DELDIVIPTIR.N	3.20	2
		1	K.EAVTVQQCYIELSK.M	4.02	2
		1	K.GTLFPM*CGM*NLAFDR.E	3.85	2
		1	K.GTLFPMCGM*NLAFDR.E	4.64	2
		1	K.GTLFPMCGMNLAFDR.E	4.58	2
		1	K.IHVPEGYDYELYNR.N	4.70	2
		1	K.KIHVPEGYDYELYNR.N	4.91	3
		1	K.KYIFTIDDDCFVAK.D	4.33	3
			R.DLIGPAM*YFGLM*GDGQPIGR.Y	4.98	2
		1	R.DLIGPAM*YFGLM*GDGQPIGR.Y	5.03	3
		1	R.DLIGPAMYFGLM*GDGQPIGR.Y	4.88	2
		1	R.EGVSTAVSHGLWLNIPDYDAPTQLVKPK.E	6.76	3
			R.YDDM*WAGWCIK.V	3.69	2
			R.YDDMWAGWCIK.V	3.37	2
			R.YVDAVM*TIPK.G	3.05	2
		1	R.YVDAVMTIPK.G	4.41	2
121	114		-.DSNGNPVKR.-	2.59	2
		1	-.EPAIIIGGER.-	2.60	2
		6	-.IEEATGAHTYK.-	2.98	2
			-.IEEATGAHTYK.-	2.79	3
			K.EPAIIIGGER.T	2.52	1
		9	K.IEEATGAHTYK.L	3.19	2
		4	K.LTTSSGTVGTIPGPWLGAPQLIATNDDAK.T	5.70	3
		40	K.LWAVDVSSSAAK.E	4.48	2
		13	K.LWAVDVSSSAAKEPAIIIGGER.T	6.45	2
			K.YFIQPAK.S	2.45	1

Table S1. *Cont.*

Spot No.	Peptide Matched Number	Dupes	Peptide Sequence	Peptide Score	Peptide Charge
		30	R.SEIWPVCNELSK.L	3.81	2
129	4		-.IEEATGAHTYK.-	2.73	3
			K.LWAVDVSSSAAK.E	3.59	2
		1	R.SEIWPVCNELSK.L	3.02	2
122	70		-.DGNDCIDVGK.-	2.47	2
			-.GSYYVLPVIR.G	2.85	2
		1	-.NGNQCPLFIGQER.-	2.56	2
			-.SAFIAVGPKPEAGGEDSSR.-	3.05	3
		1	K.FVFCRDGNDCIDVGK.N	3.83	3
		1	K.FVFCRDGNDCIDVGKNEEGGVR.G	5.55	3
			K.MDIEPTFCAQSTYWWVTTAPSPWR.S	3.55	3
		1	R.DGNDCIDVGK.N	3.55	2
		21	R.DGNDCIDVGKNEEGGVR.G	4.80	3
		15	R.GPEGGGLTLTTR.N	3.83	2
		5	R.NGNQCPLFIGQER.S	3.74	2
		10	R.SAFIAVGPKPEAGGEDSSR.S	4.80	3
		2	R.VGFVPESENLNIK.M	2.59	1
173	7		R.GPEGGGLTLTTR.N	3.66	2
		1	R.SAFIAVGPKPEAGGEDSSR.S	5.19	3
		1	R.SAFIAVGPKPEAGGEDSSR.S	5.07	2
		1	R.VGFVPESENLNIK.M	3.34	2
184	7	1	R.DGNDCIDVGK.N	2.90	2
			R.GPEGGGLTLTTR.N	3.49	2
		1	R.SAFIAVGPKPEAGGEDSSR.S	5.20	3
		1	R.VGFVPESENLNIK.M	3.40	2
125	2		K.FLDGIYVSEK.S	2.90	2
			K.HLNLDFQLIK.D	2.71	2

Table S1. *Cont.*

Spot No.	Peptide Matched Number	Dupes	Peptide Sequence	Peptide Score	Peptide Charge
168					
126	10		-.IETVDLENK.-	2.60	2
		1	K.NFKEIDEYLLK.Q	3.68	2
		1	K.TM*QVLAGDGNSPGSIR.L	5.35	2
		1	K.TMQVLAGDGNSPGSIR.L	4.98	2
			R.IETVDLENK.S	2.76	2
		1	R.LIIYGEGSPLVK.V	3.59	2
127	6	1	K.NFLSLLAENGK.L	4.45	2
		1	K.VLVTTVM*PLPPAEEK.E	4.72	2
		1	R.LAAIVDVCDK.A	3.19	2
139	27	1	K.ELKETLQEIIGEGK.K	3.65	2
			K.ELKETLQEIIGEGKK.V	4.59	2
			K.ETLQEIIGEGK.K	2.68	2
			K.ETLQEIIGEGKK.V	3.28	2
			K.FM*QLTTAHR.G	3.27	2
			K.FMQLTTAHR.G	2.49	2
		1	K.IESDLSELVEAM*K.A	4.92	2
		1	K.IESDLSELVEAM*K.A	3.67	3
			K.KFM*QLTTAHR.G	3.07	2
			K.NFLSLLAENGK.L	4.10	2

Table S1. *Cont.*

Spot No.	Peptide Matched Number	Dupes	Peptide Sequence	Peptide Score	Peptide Charge
		1	K.NFLSLLAENGK.L	3.17	1
			K.NFLSLLAENGKLK.N	2.99	2
		3	K.VLVTTVM*PLPPAEEK.E	5.02	2
		1	K.VLVTTVMPLPPAEEK.E	4.47	2
		2	R.LAAIVDVCDK.A	2.99	1
		1	R.LLREPVDLSNL.-	3.41	2
128	11		K.LIEEQETDR.G	3.01	2
		1	K.VYFDISVGNPVGK.L	4.55	2
		1	R.DFM*IQGGDFDK.G	3.27	2
		1	R.HVVFGQVIEGM*DVVR.L	4.23	2
		2	R.IVIGLFGDDVPQTVENFR.A	5.13	2
			R.IVIGLFGDDVPQTVENFR.A	4.03	3
130	14	1	K.LIEEQETDR.G	3.03	2
		1	K.VVIADCGQLPM*SEA.-	3.25	2
		1	K.VYFDISVGNPVGK.L	4.29	2
		1	R.DFM*IQGGDFDK.G	3.57	2
		1	R.HVVFGQVIEGM*DVVR.L	5.05	2
			R.HVVFGQVIEGMDVVR.L	2.05	2
		1	R.IVIGLFGDDVPQTVENFR.A	4.70	2
			R.IVIGLFGDDVPQTVENFR.A	3.37	3
134	4	1	R.IIFIGQPINAQVAQR.V	4.59	2
		1	R.VISQLVTLASIDDK.S	4.83	2
135	12		-.DRPALAM*VADAEK.-	3.81	2
		1	K.DRPALAM*VADAEKK.N	5.01	3
			K.EAEAM*KPVSVD.-	2.69	2
		1	K.RDASLLIGR.T	3.17	2
		1	R.IIM*TM*PSYTSLER.R	4.01	2

Table S1. *Cont.*

Spot No.	Peptide Matched Number	Dupes	Peptide Sequence	Peptide Score	Peptide Charge
		1	R.TPLVFLNR.V	2.84	2
		1	R.VTEGCGAYIAAK.Q	4.09	2
138	6		K.EIENGILWEVEGK.W	3.88	2
			K.LTPEQQEEFKK.G	2.74	2
		3	K.VVDIVDTFR.L	3.24	2
140	4	1	R.VNQAIFLLTTGAR.E	4.47	2
		1	K.TIAECLADELINAAK.G	5.08	2
142	6	1	K.DFPSSSSFSR.E	2.34	2
		1	K.VEIEDDSVLK.I	3.18	2
		1	R.HVEKEDKNDTWHR.V	3.80	3
143	4		K.DTDSEEELKEAFR.V	3.38	2
			K.EADVDGDGQINYDEFVK.V	5.58	2
		1	K.EAFSLFDKDGDGCITTK.E	4.28	2
		1	R.GDFAIDIGR.N	2.60	2
169	7		-.FWAIFIDEK.-	2.98	2
			K.IFGSLEAVGGAK.D	4.06	2
		1	K.LIGSWASPFSIR.A	3.50	2
		1	K.LM*ENLAILEEAFQK.S	5.38	2
			R.FWAIFIDEK.I	2.79	2
170	3		K.KKDETKPEETIDSEFEQK.V	5.36	3
		1	K.VATEESSTATGEVTDR.G	5.52	2
178	9	1	K.DHPYDPAHDSVLSR.V	3.31	2
		1	K.TNYLIVDSK.T	3.31	2
		1	K.YLFSAESVSQR.-	3.76	2
		1	R.FLVDSYNKPVPAILEIPSK.D	4.25	3
			R.KTNYLIVDSK.T	2.91	2

Table S1. *Cont.*

Spot No.	Peptide Matched Number	Dupes	Peptide Sequence	Peptide Score	Peptide Charge
179	3		K.EPAIIIGGER.T	2.6	1
		1	-.LWAVDVSSSAAK.-	3	1
183	15		-.DRNEAWIR.-	3.25	2
		1	K.GGM*IVLPAGIYHR.F	2.96	3
		1	K.GGM*IVLPAGIYHR.F	3.42	2
		1	K.KGGM*IVLPAGIYHR.F	4.63	3
		1	K.LAELGVLSWR.L	3.92	2
		1	R.FTVDSDNYIK.A	2.96	2
		1	R.LDADNYETDEELK.K	4.97	2
		1	R.LDADNYETDEELKK.I	4.21	2

Proteins have been analyzed using LC-MS/MS (LTQ XL Linear Ion Trap Mass Spectrometer, Thermo Scientific) and the acquired MS/MS data were processed for sequence identification using Mascot v 2.3 (Matrix Sciences) search engine against *Brassica* database (347,992 protein entries) downloaded from UniProtKB/Swiss-Prot with significant threshold $p = 0.05$. * represents Oxidation methionine.
